# Association of Obesity and Severe Asthma in Adults

**DOI:** 10.3390/jcm13123474

**Published:** 2024-06-14

**Authors:** Aneta Elżbieta Olejnik, Barbara Kuźnar-Kamińska

**Affiliations:** Department of Pulmonology, Allergology and Pulmonary Oncology, Poznan University of Medical Sciences, Szamarzewskiego 84 Street, 60-569 Poznan, Poland; kaminska@ump.edu.pl

**Keywords:** severe asthma, obesity, biological treatment, asthma phenotypes, bronchial thermoplasty

## Abstract

The incidence of obesity and asthma continues to enhance, significantly impacting global public health. Adipose tissue is an organ that secretes hormones and cytokines, causes meta-inflammation, and contributes to the intensification of bronchial hyperreactivity, oxidative stress, and consequently affects the different phenotypes of asthma in obese people. As body weight increases, the risk of severe asthma increases, as well as more frequent exacerbations requiring the use of glucocorticoids and hospitalization, which consequently leads to a deterioration of the quality of life. This review discusses the relationship between obesity and severe asthma, the underlying molecular mechanisms, changes in respiratory function tests in obese people, its impact on the occurrence of comorbidities, and consequently, a different response to conventional asthma treatment. The article also reviews research on possible future therapies for severe asthma. The manuscript is a narrative review of clinical trials in severe asthma and comorbid obesity. The articles were found in the PubMed database using the keywords asthma and obesity. Studies on severe asthma were then selected for inclusion in the article. The sections: ‘The classification connected with asthma and obesity’, ‘Obesity-related changes in pulmonary functional tests’, and ‘Obesity and inflammation’, include studies on subjects without asthma or non-severe asthma, which, according to the authors, familiarize the reader with the pathophysiology of obesity-related asthma.

## 1. Introduction

Asthma is a heterogeneous, chronic disease associated with inflammation of the airways, characterized by attacks of wheezing, shortness of breath, coughing, and chest tightness, which vary in time and severity [1]. It is one of the most widespread non-communicable chronic diseases. In 2019, 262 million people worldwide had asthma, and it was the cause of 455,000 deaths [2].

According to numerous studies, the incidence of severe asthma among all asthma patients is estimated at 3–10% in European countries [3,4,5,6]. According to GINA, severe asthma is defined as asthma that is uncontrolled despite high doses of ICS-LABA and optimal treatment of underlying factors, or that worsens when high doses of ICS-LABA are reduced [1]. It is estimated that severe asthma consumes about 60% of all costs related to the treatment of patients with asthma, including costs of drugs or hospitalization due to exacerbations, which is significant for public health [7]. To optimize the treatment of a patient with severe asthma, it is necessary to look for comorbidities, such as obesity, that may affect the control of asthma symptoms and the effectiveness of conventional therapies. The relevance between asthma and obesity, its impact on worsening asthma control, poorer response to treatment, changes in pulmonary functional tests, and deterioration of quality of life have been shown in numerous studies [8,9,10,11,12]. However, the exact mechanisms have not been well understood yet. Changes in the biomechanics of the chest and breathing, increased inflammation, and oxidative stress are considered to be fundament of this association.

The aim of the article, in the face of the growing increase in the incidence of asthma and obesity, is to draw attention to the co-occurrence of these two diseases and, consequently, to problems with the optimal treatment of obesity-related asthma due to its more severe course, complicated treatment regimens, and the coexistence of other obesity-related diseases. It also presents clinical trials of other possible therapies for asthma in obese people.

## 2. The Classifications Connected with Asthma and Obesity

Overweight and obesity are defined as abnormal or excessive fat accumulation that presents a risk to health [13]. The most commonly used, low cost, easy to measure, and well correlated with the health risk rate is BMI, calculated as body weight in kilograms divided by the square of height in meters [14]. For adults, the WHO defines overweight as a BMI greater than or equal to 25 kg/m^2^ and obesity greater than or equal to 30 kg/m^2^, respectively [13].

Since 1975, the number of obese people has nearly tripled to 650 million in 2016. According to the WHO data, approximately 39% of the adult population are obese, while 13% are overweight [13]. Many cross-sectional investigations have shown that asthma is more prevalent in obese compared to lean individuals [15,16,17,18]. Moreover, recent studies have demonstrated a dose–response relationship between an increased asthma incidence and an increase in BMI. Koebnick et al., in a mixed longitudinal and cross-sectional retrospective study, showed that the hazard of adult-onset asthma is increased by 12% with a BMI of 25.0–29.9 kg/m^2^, by 40% with a BMI 30.0–34.9 kg/m^2^, and by almost 250% with a BMI of 50 kg/m^2^ and above, compared to individuals with a normal weight BMI < 25 kg/m^2^ [16]. Furthermore, they proved a positive correlation between BMI and poorly controlled asthma [16]. In the Portuguese National Health Survey of 32,644 adults, overweight (25.0–29.9 kg/m^2^) individuals were associated with an odds ratio for severe asthma (defined as an emergency room visit for deterioration of asthma within the last 12 months) of 1.36, class I obesity (30.0–34.9 kg/m^2^) and class II obesity (35.0–39.9 kg/m^2^) with an OR of 1.5, and class III obesity (≥40.0 kg/m^2^) with an OR of 3.70 [9]. Holguin et al., in the study of 1049 adults from the Severe Asthma Project, evaluated obesity as a 4- to 6-fold higher risk of being hospitalized compared with lean asthmatics [19]. Moreover, in a retrospective cohort study of 72,086 patients aged 18–54 years hospitalized for asthma exacerbation, 24% were obese and were more likely to require hospitalization > 3 days, which was associated with a significantly higher risk of using mechanical ventilation compared to non-obese subjects [10].

On the other hand, the impact of weight change on asthma control, quality of life, number of courses of steroid therapy, and exacerbations was analyzed in the TENOR study (The Epidemiology and Natural History of Asthma: Outcomes and Treatment Regimens) in 2396 adults with asthma [20]. This multicenter, observational, prospective cohort study demonstrated poorer asthma control, quality of life, and a greater need for OCS in patients who gained 2.27 kg or more over 12 months of follow-up compared with patients whose weight remained unchanged or decreased by at least 2.27 kg [20].

As shown above, obesity significantly worsens asthma control, so it is worth understanding the mechanisms underlying this change. The following sections present the impact of obesity on functional pulmonary tests and the pathomechanisms of meta-inflammation caused by excess adipose tissue.

## 3. Obesity-Related Changes in Pulmonary Functional Tests

Better understanding of functional and molecular changes related to obesity is important to find associations and clinical implications, which could lead to distinct therapeutic options for obese asthmatics. Obesity causes alterations in lung mechanics independently of asthma, which determines difficulty in distinguishing physiological changes associated with obesity from those caused by asthma. Many studies have demonstrated diminution in all lung volumes, predominantly in the functional residual capacity (FRC) and expiratory reserve volume (ERV) as a result of excess adipose tissue inside thoracic cavity, around the trunk and abdomen [21,22,23,24,25,26]. Richard L Jones and Mary-Magdalene U Nzekwu showed an exponential decrease in FRC and ERV with increasing BMI, with the greatest rates of change presented in overweight and mild obesity. Moreover, they found a linear connection between BMI and vital capacity (VC) and total lung capacity (TLC); however, the group mean values remained within normal limits even among patients with obesity in class III [22]. These alterations lead morbidly obese individuals to breathe near residual volume with a low tidal volume and more frequent respiratory rates, which may contribute to their self-reported dyspnea [27]. In addition, investigations have shown a significant, uniform reduction in forced expiratory volume in one second (FEV1) and forced vital capacity (FVC), leading to a slight increase or maintenance of the FEV1/FVC ratio, which indicates a restrictive dysfunction pattern [11,21,28]. This phenomenon is probably related to the reduction in parenchymal binding forces on the attached airways, which causes expiratory dynamic closure of airways, increasing their resistance, leading to limitation of expiratory flow, air trapping, and hyperinflation [8], causing a mismatch of ventilation and perfusion [29]. All these changes occurring in the respiratory tract in obese people may cause asthma-like symptoms and, consequently, lead to an incorrect diagnosis. On the other hand, patients diagnosed with asthma may experience greater shortness of breath, and this may lead to overtreatment.

Nevertheless, despite the obesity-related reduction in peripheral airway diameter, clinical observations regarding airway hyperresponsivity (AHR) are contradictory. Many researchers have demonstrated a link between the development of AHR and increased BMI [30,31,32,33,34]. For example, Litonjua et al., in longitudinal research with 2280 men, found a non-linear association with obesity and greater AHR and a positive linear relationship between change in BMI and the subsequent development of AHR [30]. Badier et al., in a cohort study of 60 lean, 84 overweight, and 360 class I–III obese non-asthmatic individuals, noted that overweight and obese adults presented AHR about twice as often as lean individuals [31]. Moreover, weight reduction in obese asthmatics undergoing bariatric surgery resulted in an improvement in AHR [35]. Other human studies have negated this association between bronchial hyperresponsivity and higher BMI [36,37,38,39].

**Figure 1:**
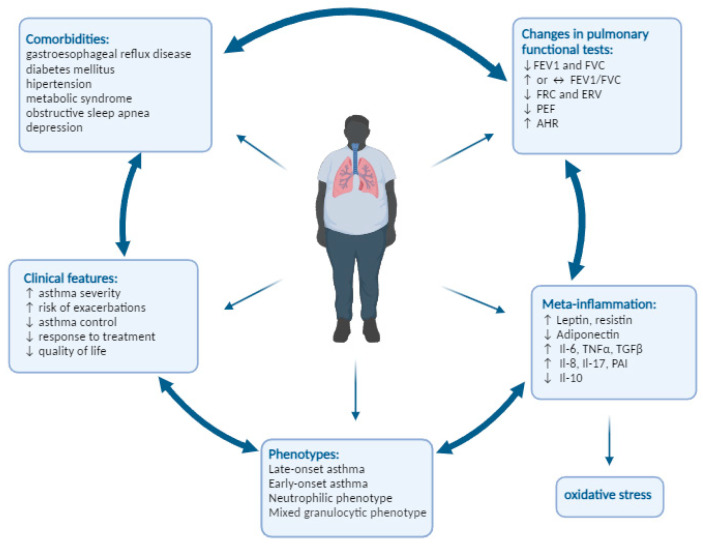
Obesity and its impact on the course of asthma. up arrow—increase; down arrow—decrease.

A summary of the changes in the respiratory system and the clinical characteristics of obesity-related asthma is presented in Figure 1.

## 4. Obesity and Inflammation

Adipose tissue both participates in energy storage and acts as an endocrine organ by secreting hormones and adipokines that modulate the immune response. Obesity causes low-grade systemic inflammation, called meta-inflammation [40]. As adipose tissue grows and the distance between adipocytes and capillaries increases, leading to hypoxia, the number of macrophages activated by fatty acids released from dead adipocytes increases [41,42]. This results in a burst of pro-inflammatory cytokines, such as IL-6, TNF-α, IL-1β, and leptin [40,42], which drain from adipose tissue via portal circulation to the pulmonary vasculature and, consequently, may affect the development and course of asthma [35]. The most important cytokines and their impact on asthma are presented in a Table 1.

**Figure 2:**
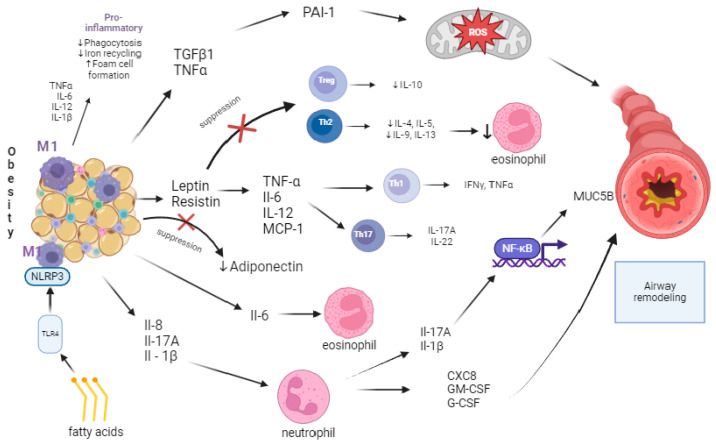
Pathophysiology of airway remodeling related to cytokine secretion by adipose tissue. up arrow—stimulation of cytokine/cell production; down arrow—suppression of cytokine/cell secretion.

A schematic summary of the pathophysiology of airway remodeling induced by meta-inflammation present in obesity is shown in Figure 2.

## 5. Clinical Characteristics of Asthma in Obesity

### 5.1. Endotypes and Phenotypes

Taking into account the pathophysiological mechanisms at the cellular and molecular levels, there are two main endotypes of asthma: Th2 high and Th2 low [121]. In the high Th2 endotype, eosinophilic and mixed subtypes can be distinguished, while in the low Th2 endotype—neutrophilic and paucigranulocytic. All these subtypes, except paucigranulocytic, may occur in severe asthma. Moreover, recent studies based on cluster analysis of obesity-related asthma have identified two distinct phenotypes: late-onset asthma due to obesity and early-onset asthma, in which pre-existing symptoms worsen with weight gain [122,123]. Understanding the characteristic features of individual endotypes and phenotypes is important in selecting appropriate, effective asthma therapy, and these are presented in the sections below.

#### 5.1.1. Late-Onset Asthma (LOA)

Haldar et al., in the cluster analysis, identified a group of predominantly obese women (81.5%) with later onset of asthma (35 ± 19 years), reduced atopy, and a higher level of symptoms’ expression in the low Th2 inflammation (decreased blood and sputum eosinophils, and less FeNO) and enhanced neutrophilic inflammation [122]. Similar observations were performed by Moore et al. within the study of 726 subjects with persistent asthma from the NIH Severe Asthma Research Program (SARP). They distinguished a LOA phenotype (all had asthma diagnosed > 23 years), predominating in older women (mean age 50) with the highest BMI (58% > 30 kg/m^2^) and lower baseline lung function (71% of FEV1 < 80% predicted), despite the relatively short duration of the disease. Compared to other clusters, the lowest bronchial hyperresponsiveness and reduced atopy were characteristic for that group (64% of patients had ≥1 positive SPT and lower total IgE) [123].

In a comparative study for both phenotypes of obesity-related asthma, obese individuals with LOA predominated in severe disease and required more complicated treatment regimens [19]. Moreover, regardless of using ≥ 3 asthma control medications, including high-dose ICS (54% of patients) and more frequent use of oral steroids (17% of patients used them regularly, 36% required ≥ 3 OCS, burst/year), they reported greater daily symptoms and high care utilization associated with exacerbations. The representatives of this cluster were also more likely to have comorbidities, such as sinus disease, gastroesophageal reflux, and hypertension [123].

#### 5.1.2. Early-Onset Asthma (EOA)

In a cluster analysis, Haldar et al. [122], in addition to LOA, distinguished a group of predominantly male patients (54%) with early-onset asthma (14.6 ± 15.5 years), which was characterized by atopy (95%), eosinophilic inflammation (higher percentage of eosinophils in sputum, and increased FeNO), more frequent exacerbations requiring oral corticosteroids, and hospitalization, compared to other clusters treated in primary care. In secondary care, this phenotype was dominant in females (75.7%) and was characterized by a lower quality of life due to poorer control of symptoms, greater eosinophilic inflammation, and airway obstruction, despite the use of the highest doses of ICS and more frequent treatment with oral glucocorticoids [122].

In a study comparing LOA and EOA, the latter showed greater airway obstruction, which was positively correlated with BMI, greater bronchial hyperresponsiveness, and elevated IgE. Moreover, obese EOA individuals had a higher risk of pneumonia and exacerbation-related hospitalization, ICU admission, and mechanical ventilation compared with obese LOA subjects. Obese patients with EOA were three and six times more likely to be hospitalized and admitted to the ICU than their lean counterparts [19].

Furthermore, representatives of this phenotype showed a linear increase in BMI with the duration of asthma, also after taking into account its severity. This phenomenon was not observed among LOA subjects. The results may suggest that in early-onset asthma, pre-existing symptoms worsen with weight gain, whereas in late-onset asthma, the severity of asthma and BMI may be due to a cause-and-effect relationship [19].

#### 5.1.3. Neutrophilic Phenotype of Severe Asthma

The most common manifestation of severe asthma seen in adults, with onset predominantly > 12 years of age [123,124], is neutrophilic asthma, characterized by a sputum neutrophil count of 500 × 10^4^/mL [125] or 40% to 76% of all sputum cells [124,125,126,127], with a reduced eosinophil count (usually less than 1.9% to 3%) [122,128]. However, sputum analysis has limited availability and requires advanced training, which makes its clinical use difficult. The diagnosis of neutrophilic asthma can also be complicated by high doses of inhaled or oral steroids that increase neutrophil counts in tissues and peripheral blood [129].

Previous studies have shown that the neutrophilic phenotype is more likely to occur in obese than in lean women (42.9% vs. 16.2%) [130], non-atopic [123,131], with persistent remodeling airway obstruction [127,132,133] and lower AHR compared with eosinophilic asthma [123,131].

Furthermore, it was associated with poorer quality of life and prognosis [123,128,134,135] due to more severe steroid-resistant asthma [136,137], poorer symptom control [123] with recurrent nocturnal attacks [138], frequent non-infectious exacerbations requiring urgent emergency room visits, hospitalization, and mechanical ventilation [139,140]. A higher risk of exacerbations may also be associated with chronic atypical bacterial inflammation, as seen in patients with severe neutrophilic asthma [141].

#### 5.1.4. Mixed Granulocytic Asthma

Scott proposed distinguishing the obesity-related neutrophilic asthma as a subgroup of late-onset asthma, but it may also include patients with mixed granulocytic inflammation [130]. Moore et al. [123], in cluster 5, observed a group of mostly elderly (49 ± 11 years), obese (51% with a BMI > 30 kg/m^2^) women (63%) with late-onset asthma (21 ± 15 years) and longer duration (29 ± 15), accompanied by mixed inflammation: eosinophilic and neutrophilic. This phenotype was also characterized by less atopy (66% of patients had ≥1 positive SPT), greater airway limitation (FEV1 pred. 43 ± 14%), more severe disease manifested by complicated treatment regimens (95% taking ≥2 and ≥3 controller drugs were taken by 95% and 67%, respectively), and frequent use of high-dose ICS and oral corticosteroids. Moreover, representatives of this group had worse disease control, with recurrent exacerbations requiring oral steroid bursts, admission to ED, or hospitalization, including ICU [123]. Further research is needed to improve the clinical characteristics of this mixed granulocytic endotype [130].

A summary of the endotypes and phenotypes of obesity-related asthma is presented in Table 2.

### 5.2. Comorbidities

More than mild-to-moderate, severe asthma predisposes to various pulmonary and extrapulmonary comorbidities [142,143]. Obesity, together with female gender, older age, former smoking, or steroid resistance, are factors of their more frequent occurrence [144]. Due to the significant impact of comorbidities on the course and severity of asthma [143], prompt diagnosis and appropriate treatment are crucial to improve asthma and avoid overtreatment. Below, the diseases comorbid with asthma and obesity are presented, the appropriate treatment of which significantly improves the control of asthma symptoms.

#### 5.2.1. Chronic Rhinosinusitis and Nasal Polypus

Most asthmatics have symptoms of seasonal or perennial allergic rhinitis [142]; therefore, these two entities are considered to constitute one complex syndrome of asthma and allergic rhinitis, characterized by Th2 inflammation [145]. Rhinitis is also common in non-allergic asthma [146]. Chronic rhinosinusitis (CRS) occurs in 1/3 of patients with severe asthma [147], predominantly neutrophilic (>64%) [148], and 20% additionally have nasal polyps [149]. The evidence has shown that chronic rhinosinusitis worsens asthma symptom control, increases the frequency of exacerbations, and impairs quality of life [150], while medical and surgical treatment improves the clinical course of severe asthma [142]. The use of monoclonal antibodies, including omalizumab, mepolizumab, and dupilumab, in the treatment of CRS with polyps is promising, which in randomized clinical trials has been shown to be effective in reducing the size of polyps and, consequently, symptoms [151].

#### 5.2.2. Obstructive Sleep Apnea (OSA)

Both obesity [152] and severe asthma increase the risk of obstructive sleep apnea, with an odds ratio of 4.36 for severe and difficult-to-control asthma [153]. OSA was more likely to occur in the neutrophilic phenotype and was associated with more self-reported asthma symptoms, more frequent use of β2-agonists [154], and a higher risk of exacerbations [155]. There is evidence that treating OSA with continuous positive airway pressure (CPAP) reduces systemic inflammation and asthma symptoms, thereby improving both asthma control and obesity [156,157].

#### 5.2.3. Gastroesophageal Reflux Disease (GERD)

The prevalence of GERD in patients with asthma increases with severity and accounts for 21% and 46–63% of mild-to-moderate and severe asthma, respectively [142]. Moreover, obesity was associated with a higher incidence of GERD among patients with severe asthma [123]. Gastric acid micro-aspirations present in reflux contribute to increased bronchoconstriction by activating the vagus response [158] and increasing AHR indirectly by inducing airway chronic neutrophilic inflammation [159], which may lead to poorer asthma control and more frequent exacerbations [142]. On the other hand, bronchodilators, including beta-agonists, anticholinergics, and methylxanthines, reduce the tone of the lower esophageal sphincter, worsening reflux symptoms [160]. Evidence has demonstrated conflicting results regarding clinical improvement of asthma with proton pump inhibitors (PPIs), with most showing little or no treatment effect, although none of the studies focused on obese asthmatics [157].

For this reason, GINA does not recommend the systematic use of PPIs in uncontrolled asthma with asymptomatic GERD [1]. In addition, the evidence supporting the effectiveness of surgery in adults with GERD and concomitant asthma is currently insufficient [161].

In summary, it seems clinically important to treat GERD with proton pump inhibitors only when symptoms of reflux disease occur.

#### 5.2.4. Diabetes Mellitus Type 2 and Metabolic Syndrome

Abdominal obesity is one of the components of metabolic syndrome (MetS), and the others include: dyslipidemia (hypertriglyceridemia or low HDL), hyperglycemia or treated diabetes, and elevated blood pressure > 130/85 mmHg or treated hypertension, and ≥3 of the above features must be present to be diagnosed [162]. The incidence of MetS is high in asthmatic patients; however, there was no significant relationship between the incidence of the syndrome and the severity of asthma [163,164]. Asthmatics with metabolic syndrome after bariatric surgery had less improvement in asthma control compared to those without metabolic syndrome, which may be due to greater systemic inflammation caused by metabolic dysregulation [165].

Lipid disorders often accompany obesity [162]. A study of 85,555 people found that wheezing was positively related to BMI, waist circumference, a high TG level, high blood pressure, and metabolic syndrome, but inversely related to s-HDL [166]. Moreover, it has been observed that low HDL levels in childhood may be associated with an increased risk of asthma in adolescence [167]. Previous analyses found no effect of statins on improving asthma [166]; however, a more recent meta-analysis of 11 randomized and 8 observational studies showed improved asthma control on the ACT and ACQ questionnaires and a reduction in asthma-related ED visits in patients taking statins compared to placebo. No improvement in lung function (FEV1 and PEF) was observed [168].

There is evidence that asthma was associated with a 31% higher risk of DMt2 (OR = 1.31); however, this relationship weakened when BMI was adjusted for (OR = 1.25) [169], suggesting that chronic inflammation is involved in the pathogenesis. Insulin resistance, a characteristic feature of obesity, may also contribute to worsening of asthma by blocking M2 muscarinic receptors, and increasing the release of acetylcholine from parasympathetic airway neurons causes bronchospasm [170]. In addition, due to higher use of OCS and insulin resistance, severe asthma is associated with an enhanced incidence of diabetes compared to mild and moderate asthma [171]. Concomitant diabetes worsens asthma control. Studies have shown a positive correlation between Hb1Ac levels and the risk of exacerbation [172] and hospitalizations [173]. Hyperglycemia was associated with longer hospital stays due to exacerbations [174] and higher mortality [175]. Moreover, taking metformin reduced the risk of asthma exacerbations and hospitalization [176].

The use of glucagon-like peptide receptor 1 (GLP-1R) analogues seems promising, as lower rates of asthma exacerbations and symptoms have been observed compared to other diabetic treatment [177]. This may be related to their effect on weight loss, improvement of insulin sensitivity, and reduction of systemic inflammation [178]. In an obese mouse model of asthma, the GLP-1R agonist reduced both eosinophilic airway inflammation through a decrease in the cytokines IL-4, 5, and 33, and neutrophilic inflammation through inhibition of NLRP3 inflammasome activity and suppression of IL-1β [179]. In addition, a study using isolated human bronchi found GLP-1 receptors in the airway epithelium, mucous glands, inflammatory cells, and ASM of the mid-bronchial airways and observed a positive effect of GLP-1 agonists in modulating ASM contraction and improving AHR [180]. Further investigations are needed to evaluate the benefits of using GLP-1 agonists in the treatment of asthma with comorbid obesity or diabetes.

In conclusion, treating obesity as one of the components of metabolic syndrome is important not only to improve asthma control and quality of life, but it may also reduce or eliminate other diseases that are part of metabolic syndrome. The use of GLP-1 analogues seems particularly promising in the therapy of diabetes and obesity.

## 6. Influence of Obesity on Asthma Management

In addition to exacerbating self-reported symptoms and worsening asthma control, obesity also affects the response to controller medications. A review of clinical trials on the effectiveness of conventional treatments for obesity-related asthma is discussed below.

### 6.1. Montelukast

Peters-Golden et al. showed a negative correlation between asthma control days (ACD) and increasing BMI, while the response to montelukast, as measured by ACD, was stable across all BMI categories, also after adjusting for placebo [181]. This phenomenon may be due to the leptin-stimulated regulation of the production of leukotrienes, as demonstrated in studies on rats [182]. In human observations, a positive correlation has been seen between BMI and an increase in leukotriene synthesis [183]. On the other hand, a retrospective study comparing the response to treatment with fluticasone propionate plus salmeterol and montelukast alone showed higher FEV1 and better asthma control in the FP/SAL arm than montelukast across the BMI range [184]. Further research is needed to determine the role of montelukast in the treatment of obesity-related asthma.

### 6.2. ICS and LABA/LAMA

Obese asthmatics, compared to lean individuals, are more likely to have severe persistent asthma, a significantly lower chance of achieving well-controlled asthma [185] despite the use of higher doses of ICS [186] and, therefore, more frequent use of controller medications, including ICS, LABA, and oral GCs [185]. Obesity was also associated with a worse response to ICS and ICS + LABA in terms of FEV1 and the FEV1/FVC ratio, as well as less improvement in FeNO levels after ICS [187]. In addition, Camargo et al. showed that obese asthmatics required longer treatment with ICS+LABA to achieve peak FEV1 compared to their lean counterparts [184]. Additive tiotropium therapy improved peak FEV1 and trough FEV1 across all BMI categories in both moderate and severe symptomatic asthma patients; however, the most significant increase in FEV1 was observed in the former [188].

There is also evidence of significant differences in response to ICS treatment between eosinophilic and neutrophilic phenotypes, which can be used as a predictive factor. McGrath et al. showed an improvement in airway obstruction only in a group of patients with eosinophilic asthma after two weeks of intensive combined anti-inflammatory therapy with prednisone, inhaled budesonide, and zafirlukast [189]. Similar results were obtained in another study where obese patients with uncontrolled eosinophilic asthma responded well to an increase in ICS, while their non-eosinophil/neutrophil counterparts experienced a worsening of control after intensification of ICS treatment [190].

Steroid hypo-responsiveness in the neutrophilic phenotype of obese asthmatics may be related to dysregulation of glucocorticoid receptors. It has been shown that the cytokine IL-17, the concentration of which increases in obesity and responses to neutrophil inflammation, upregulates the glucocorticoid-beta receptor (GR-β), which is a negative regulator of active GR-α [191]. In addition, a decrease in the GRα/GRβ ratio was observed after incubation of adipocytes taken from obese patients in the IL-17A environment, compared to adipocytes obtained from lean individuals whose GR-α/GR-β ratio was enhanced after stimulation with IL-17A and IL-17F [192].

Another possible mechanism of GCS resistance is the reduced induction of MAP kinase phosphatase-1 gene expression in PBMC and BAL cells in response to dexamethasone, associated with enhanced TNF-α synthesis, which significantly decreased with increasing BMI. This effect was not seen in the non-asthmatic control group [193].

### 6.3. Biological Treatment

For two decades, a new biological therapy has been available, mainly aimed at the treatment of Th2 inflammation, including anti-IgE agents, such as omalizumab; antibodies directed against IL-5 receptors or anti-IL-5, such as benralizumab, mepolizumab, and reslizumab; anti-IL-4Rα agents, such as dupilumab, and anti-thymic stromal lymphopoietin antibodies, such as tezepelumab [1]. However, conventional Th2 inflammation biomarkers are altered in obesity, with an inverse relationship between FeNO and BMI associated with increased oxidative stress in obese individuals [194]. In addition, poor correlation was observed between serum eosinophilia, total IgE, FeNO, and sputum eosinophilia [195]. This may cause difficulties in the diagnosis of Th2 inflammation in obese asthmatics, and thus delay the implementation of appropriate biological treatment.

Concerning the heterogeneity of pathogenesis of the obesity-related asthma, it might be expected that the biological treatment may not be as effective in obese asthmatics; however, the evidence is conflicting. GuC et al. [196] showed an improvement in asthma control, as measured by the Asthma Control Test (ACT), after omalizumab administration in all BMI groups, but they observed that the BMI among responders was significantly lower than among non-responders, most of whom were obese. Moreover, an increase in FEV1% predicted and FVC% predicted was found only in non-obese patients [196]. Similar observations were performed on a larger group of 340 people by Sposato et al., noting a reduced response of omalizumab to FEV1, FVC, ACT, and FeNO in obesity [197]. In contrast, a prospective study of patients treated for 12 months with omalizumab demonstrated significant improvements in asthma control, lung function, a reduction in the daily dose of budesonide, and statistically significant weight loss, but the evidence was limited due to the small cohort (32 subjects, including 19 obese) [198]. Furthermore, omalizumab dosing is individualized based on serum IgE concentration and body weight according to a specific drug registration table, which may be problematic in obese patients with high IgE concentrations, as the lack of sufficient data precludes dosing in this group of patients [199].

Another biological drug dosed depending on body weight is reslizumab. Unfortunately, there is still no data on the relationship between its effectiveness and BMI. In an observational study of 134 patients with severe eosinophilic asthma, 30.5% of whom were obese, only a minority of patients (13.6%) did not improve with reslizumab, while the rest showed reduced asthma exacerbations, use of OCS, and reliever medication, and improved lung function [200].

Conflicting reports also apply to mepolizumab. The DREAM study in cluster 4 of patients with the late-onset asthma phenotype (mean age of diagnosis 32 years, with the highest BMI, number of exacerbations, and airway reversibility, and the lowest FEV1) showed a 67% diminution in exacerbations, significantly greater than non-obese counterparts in other clusters [201]. On the contrary, a more recent post hoc analysis of data from the Phase IIb/III trials DREAM, MENSA, SIRIUS, and MUSCA showed a reduction in exacerbation rates and improvements in lung function, asthma control, and quality of life notwithstanding comorbidities, including obesity [202].

Regarding the anti-IL5 receptor antibody, a poorer response to benralizumab was observed in reducing annual exacerbations in patients with a BMI > 35 kg/m^2^ [203]. Interestingly, in the study comparing reslizumab, mepolizumab, and benralizumab, a statistically significant decrease in body weight was demonstrated after six months of all the above treatments, more pronounced in people with an initial BMI > 30 kg/m^2^. This may have been related to the reduction in exacerbations and the use of OCS; however, due to the small sample size, the clinical relevance of this study is unclear [204].

A post hoc analysis of the QUEST phase III study of dupilumab, including 1584 patients with elevated biomarkers of Th2 inflammation, showed a reduction in the annual rate of asthma exacerbations, regardless of demographics, such as age of onset or BMI, with the greatest treatment effects in those with higher serum eosinophils and FeNO [205].

The latest therapy registered by the European Union in September 2022 is an antibody against thymic stromal lymphopoietin (TSLP), a cytokine that is involved in both driving Th2 inflammation and Th2-independent mechanisms [206]. Its concentration is positively correlated with the severity of asthma, the degree of airway obstruction, and resistance to glucocorticoids [206,207]. The PATHWAY and NAVIGATOR studies have demonstrated a reduction in the annual rate of exacerbations, blood eosinophils, FeNO, and IgE, and improvement in FEV1 and quality of life, regardless of the baseline blood eosinophil level (also in the group < 300 cells/µL), which may be a promising treatment in the non-eosinophilic asthma phenotype [208,209]. Unfortunately, there are no large studies assessing the effects of tezepelumab in patients with obesity-related asthma.

A summary of clinical studies on the effectiveness of conventional treatments for obesity-related asthma is provided in Table 3.

## 7. Other Approaches to Severe Asthma Management

Current treatment strategies for asthma in obese patients are mainly based on maximizing therapy with inhaled glucocorticoids and bronchodilators. In severe asthma, biological treatment and the use of systemic glucocorticoids are also possible. Targeted treatment strategies for obesity-related asthma are still lacking. The research results of possible other treatments for severe asthma are presented below and summarized in Table 4.

### 7.1. Macrolide Antibiotics

In the guidelines, GINA recommends considering adding azithromycin to ICS-LABA therapy in persistent symptomatic asthma, administered three times a week for at least six months, after checking the status of mycobacteria in the sputum [1]. Evidence from the AMAZES study showed that in patients with both eosinophilic and non-eosinophilic asthma on medium to high doses of ICS plus LABA, azithromycin reduced exacerbation rates and improved quality of life [210]. In contrast, in the AZISAST study, a diminution in the risk of exacerbations and lower respiratory tract infections was observed only in the severe non-eosinophilic asthma, while an improvement in quality of life was reported in both phenotypes [211]. Macrolides, apart from anti-infective activity, also have anti-inflammatory effects: they inhibit the activation of NF-ĸB and secretion of IL-8 [234], promote phagocytosis [235], and suppress the dysregulation of the TNF pathway [236], contributing to the reduction of neutrophilic inflammation [212].

### 7.2. Roflumilast

Roflumilast is a type 4 cAMP phosphodiesterase inhibitor included in the treatment regimen for chronic obstructive pulmonary disease. However, its anti-inflammatory effect, inhibiting subepithelial fibrosis and ASM hypertrophy, and thus positively affecting remodeling of the airways and reducing AHR, may be used as an additive therapy in asthma [237]. Evidence has shown that roflumilast reduces both eosinophilic and neutrophilic allergen-induced inflammation [213], and in combination with montelukast, improves lung function and symptom control in mild-to-moderate, inadequately controlled asthma. However, this study did not evaluate efficacy in moderate-to-severe asthma, which would be worth investigating in future studies [214].

### 7.3. Bronchial Thermoplasty

An alternative form of treatment for adults with severe asthma that remains uncontrolled despite optimal treatment with high doses of ICS + LABA, available in some countries and included in the GINA guidelines, is bronchial thermoplasty (BT) [1]. This is a method aimed at reducing ASM hypertrophy, consisting of three sessions of targeted local radiofrequency pulse performed during bronchoscopy at three- to four-week intervals in specialized centers [238]. Patients qualified for this procedure should be in a stable period of the disease, without exacerbations within two weeks before the procedure and recurrence of lower respiratory tract infections a year before BT, due to the increased risk of infection and deterioration of the condition and hospitalization during the three-month treatment period. Despite these limitations, studies evaluating the safety and effectiveness of BT have shown that this procedure was associated with significant improvements in ACQ and AQLQ [228,229], and a reduction in the incidence of severe exacerbations and emergency department visits [229,230,231,232]. However, data regarding the increase in FEV1 are contradictory [228,229,230,231,232].

A recent study assessing the effect and safety of BT ≥ 10 years after BT, which enrolled 192 of 429 patients from the AIR, RISA, and AIR2 research, showed a similar frequency of severe exacerbations at the BT10+ visit as at one year post-procedure [233]. Likewise, quality of life and lung function were similar at one, five, and ten years after BT. In the AIR2 study, an additional HRCT examination was performed, which showed that six (7%) participants were diagnosed with new bronchiectasis (five participants mild and one moderate) [233].

In summary, bronchial thermoplasty may be considered in special cases of patients with severe asthma, uncontrolled by conventional treatment methods, performed in selected centers with experience in its implementation.

## 8. Conclusions

Asthma and obesity are two diseases whose coexistence aggravates each other’s course. Their incidence is constantly increasing, posing a serious burden to public health. Our understanding of the pathogenetic mechanisms of asthma and obesity and their interaction is expanding, but there are still many unexplored areas. Future research should focus on better detailing the clinical and molecular features of obesity-related asthma phenotypes and finding specific biomarkers, especially in low Th2 inflammation. Despite the paradigm of the increasingly popular approach to treatment according to precision medicine and the observed lower effectiveness of anti-asthmatic drugs in obese patients, individualized treatment regimens for obesity-related asthma are still lacking. This is certainly a challenge for the future.

## Figures and Tables

**Table 1:** Chemokines and cytokines secreted by adipose tissue, their systemic effects, and their impact on the respiratory system.

Adipokine	Systemic Effect	Effect on Asthma
Leptin	▪ is a mediator of appetite and energy expenditure—anorexigenic effect (inhibits hunger) [43] ▪ is increased in obesity, aggravates insulin resistance [44] ▪ stimulates adipocytes to secrete pro-inflammatory cytokines, such as TNF-α, Il-6, IL-12 [45], and MCP-1 [46] ▪ facilitates phagocytosis [45] ▪ activates peripheral blood mononuclear cells (PBMC) to induce the secretion of Il-6 and TNF-α [47,48] ▪ enhances the formation of oxygen radicals in neutrophils [49] ▪ promotes the production of T lymphocytes and their differentiation toward cells producing cytokines for Th1, inhibits the functions of regulatory Treg lymphocytes [50], and affects the activity of pro-inflammatory Th17 cells [51]	▪ leptin and leptin receptor are expressed in the airway epithelium [35,52] ▪ leptin level is significantly higher in obese asthmatics compared to obese controls and is associated with greater AHR [35] ▪ is positively associated with asthma severity, asthma control, and lung function [53]
Adiponectin	▪ is involved in the stimulation of food intake (anorexigenic hormone) [54] ▪ is decreased in obesity [55] ▪ negatively correlates with insulin resistance by activating AMPK in muscle and liver [54] ▪ stimulates expression of anti-inflammatory cytokines, such as IL-10, an endogenous antagonist of the IL-1 receptor [56] ▪ inhibits production of pro-inflammatory cytokines TNF-α, IL-6, and INF and the expression of the transcription factor NF-κB in macrophages [56,57] ▪ promotes the differentiation of monocytes into M1 macrophages and inhibits differentiation into M2 macrophages [58]	▪ adiponectin receptors (adipoR1, adipoR2, and T-cadherin) are present in bronchial airways, lung parenchyma, and alveolar fluid [54,59] ▪ reduces AHR in lean people; in obese people, lowering the concentration of adiponectin leads to an increase in AHR [60] ▪ inversely relates with asthma control [61]
Resistin	▪ is increased in obesity [62] ▪ induces insulin resistance [63] ▪ stimulates macrophages to produce TNF-α and IL-12 [64] ▪ plays a role in the differentiation of monocytes and macrophages [65]	▪ augments mucin expression in airway epithelial cells [66] ▪ plays a role in a dose-dependent effect on asthma severity [67] ▪ induce airway remodeling [68]
TNF-α	▪ serum concentration of TNF-α is elevated in obesity [69] ▪ induces insulin resistance by reducing the expression of insulin-regulated glucose transporter type 4 and by impairing the insulin signaling through serine phosphorylation [70] ▪ causes an increase in the level of reactive oxygen species (ROS) because of mitochondrial dysfunction [71] ▪ stimulates PAI-1 expression in the adipose tissue [72]	▪ In normal subjects, it induces AHR and augments neutrophil infiltration to airways [73] ▪ expression of membrane-bound TNF-α, TNF-α receptor 1, and TNF-α-converting enzyme by PBMC in patients with refractory asthma [74]
TGF-β1	▪ promotes the browning of white adipose tissue [75] ▪ induces PAI-1 secretion [72]	▪ is positively associated with asthma severity [76] ▪ contributes to upregulation of PAI-1 expression in airway epithelial cells [77]
PAI-1	▪ is elevated in obesity and associated with insulin resistance [72,78] ▪ plays a key role in proinflammatory, profibrotic, and mitogenic mechanisms [79]	▪ contributes to an increase in AHR [80,81] ▪ plays a role as a mediator of airway inflammation and remodeling [81,82]
	▪ inhibits tissue plasminogen activator (tPA) and urokinase (uPA), which suppress fibrinolysis [83] ▪ elevated level of PAI-1 plays a key role in atherosclerosis and cardiovascular disease, thrombosis, tumor invasion, and metastasis [84]	
IL-6	▪ positively correlates with BMI [85] ▪ mediates the suppression of food intake by stimulating the glucagon-like peptide-1 (GLP-1) receptor [86] ▪ increases insulin secretion by stimulating the production of GLP-1 [86] ▪ stimulates neutrophils to produce IL-17 [87] ▪ adipose tissue-derived dendritic cells (ATDCs) express higher levels of IL-6, TGF-β, and IL-23, which promote differentiation of Th17 cells [88]	▪ associates with diminished FEV1 [89], more frequent asthma exacerbations [89,90], blood eosinophilia, submucosal infiltration of T-cells, and macrophages [90]
IL-17A	▪ is elevated in obesity [91]; IL-17A receptor C (IL-17RC) is expressed in mature adipocytes [92] ▪ induces production of TNF-α, IL-6, and IL-1β [93] ▪ plays a key role in pathogenesis of lupus, psoriasis, rheumatoid arthritis, multiple sclerosis, inflammatory bowel disease, and cancers [94,95]	▪ increases the influx of neutrophils into airways by affecting the synthesis of chemokines (CXC) and growth factors G-CSF and GM-CSF [96] ▪ airway neutrophilia and sputum level of IL-17 positively correlates with asthma control and negatively correlates with FEV1 and serum IL-17 levels [97] ▪ induces MUC5B expression in bronchial epithelial cells via the NF-κB pathway, which affects airway remodeling [98]
IL-1β	▪ is a major endogenous pyrogen [99] ▪ is increased in obesity [100] ▪ induces impaired insulin signal transduction by reducing the expression of signaling proteins and glucose transport proteins (GLUT4) [101] ▪ fatty acids via toll-like receptor 4 (TLR4) activate the inflammasome of nucleotide oligomerization domain-like receptor protein 3 (NLRP3), which then activates caspase-1, resulting in release of IL-1β and neutrophil influx [12] ▪ affects the activation of T lymphocytes, proliferation of B cells, and increasing the synthesis of immunoglobulins [102] ▪ induces cyclooxygenase-2 [102]	▪ the level of IL-1β in the sputum positively correlates with neutrophilic inflammation and obesity by enhanced expression of the NLRP3 genes [103] ▪ plays a key role in airway remodeling by regulating airway smooth-muscle responses [104,105], increasing AHR [105], and inducing MUC5B expression in bronchial epithelial cells via the NF-κB pathway [98] ▪ is associated with an increased risk of asthma exacerbations [106] ▪ positively correlates with asthma severity [106]
IL-8	▪ is increased in obesity [100,107] ▪ promotes insulin resistance by inhibition of insulin-induced Akt phosphorylation in adipocytes [108] ▪ activates neutrophils [109] ▪ induces the migration of cells and fibroblasts to the wound, and hence promotes healing [110] ▪ supports angiogenesis [111] ▪ plays a key role in pathogenesis of psoriasis rheumatoid arthritis, chronic obstructive pulmonary disease, endometriosis, inflammatory bowel disease, development of cancers, and signaling in cystic fibrosis [112]	▪ correlates with number of neutrophils in BALF and the level of myeloperoxidase (MPO), which proves the role of IL-8 in inducing neutrophil chemotaxis to the airways [113] ▪ IL-8 gene and protein expression is upregulated in the bronchial epithelium of patients with symptomatic asthma [114] ▪ positively correlates with asthma severity [115] ▪ an increase in IL-8 sputum levels precedes an exacerbation of asthma [116]
IL-10	▪ is negatively correlated with BMI [117] ▪ inhibits adipogenesis in preadipocytes [118] ▪ is associated with insulin sensitivity [117] ▪ is a major anti-inflammatory cytokine that reduces TNF-α, IL-6, and IL-8 [119]	▪ IL-10 level in sputum is significantly lower in asthmatic individuals [120]

**Table 2:** Asthma phenotypes in obesity and a summary of their characteristic features.

Late-Onset Asthma Phenotype	Early-Onset Asthma Phenotype	Neutrophilic Phenotype	Mixed Granylocytic Phenotype
Older women Less Th2 inflammation Less atopy Low baseline lung function Frequent exacerbations More complicated treatment regimens: high doses of ICS, more frequent use of OCS Frequent sinus disease, GERD, hypertension	Young men in primary care, young women in secondary care Atopy High eosinophilic inflammation Greater airway obstruction and AHR Persistent treatment with OCS More frequent hospitalization due to exacerbations	Most common phenotype in severe asthma Older woman Longer asthma duration Less atopy Neutrophilic inflammation Chronic atypical bacterial inflammation Persistent remodeling airway obstruction Steroid resistance Recurrent nocturnal attacks, frequent non-infectious exacerbations	Older women Longer asthma duration Mixed inflammation: eosinophilic and neutrophilic Less atopy Greater airway obstruction Frequent exacerbations More complicated treatment regimens: high doses of ICS, more frequent use of OCS

**Table 3:** Summary of clinical trial results regarding the effectiveness of conventional methods of treating asthma with comorbid obesity.

Therapy	Author	Study Characterictic	Main Findings
Montelukast	Peters-Golden et al. [181]	Post hoc analysis of 4 randomized double-blind, placebo-controlled studies of 3073 moderate asthmatic adults	Response to montelukast, as measured by ACD, was stable across all BMI categories
Montelukast vs. fluticasone propionate/salmeterol	Camargo et al. [184]	Retrospective meta-analysis of 4 clinical trials	Higher FEV1 and better asthma control were observed in the fluticasone propionate/salmeterol arm than montelukast across the BMI range
ICS	Mc Grath et al. [189]	Post hoc analysis of 9 clinical trials of 995 subjects with persistent asthma	Improvement in airway obstruction was observed only in the group of patients with eosinophilic asthma after 2 weeks of intensive combined anti-inflammatory therapy with prednisone, inhaled budesonide, and zafirlukast
ICS + LABA	Camargo et al. [184] Taylor et al. [185] Sutherland et al. [187]	Retrospective meta-analysis of 4 clinical trials Retrospective analysis from the National Asthma Survey of 3095 patients with asthma Retrospective analysis from Asthma Clinical Research Network studies of 1265 patients with mild-to-moderate persistent asthma	Obesity was associated with poorer response to ICS and ICS + LABA in terms of FEV1 and FEV1/FVC ratio and less improvement in FeNO levels after ICS. Obese asthmatics required longer ICS + LABA treatment to reach peak FEV1 compared to their lean counterparts.
ICS + LABA + LAMA	Khurana et al. [188]	Post hoc analysis from 5 phase III clinical trials of tiotropium soft mist inhaler in patients with differing severities of asthma (912 of 3476 had severe asthma)	The addition of tiotropium to ICS+LABA therapy improved peak and trough FEV1 across all BMI categories in both moderate and severe asthma patients.
Omalizumab	GuC et al. [196]	Comparative study (obese vs. non-obese) of 45 patients with moderate-to-severe uncontrolled asthma	Improved asthma control, as measured by the Asthma Control Test (ACT), in all BMI groups
Omalizumab	Sposato et al. [197]	Real-life retrospective study of 340 patients with severe asthma	Reduced response of omalizumab to FEV1, FVC, ACT, and FeNO in obesity was noticed
Omalizumab	Oliveira et al. [198]	Prospective study of 32 subjects with severe asthma	Significant improvement in asthma control, lung function, reduction in the daily dose of budesonide, and statistically significant weight loss
Reslizumab	Hashimoto et al. [200]	Observational real-world study of 134 adults with severe eosinophilic asthma	Study of patients with severe eosinophilic asthma, 30.5% of whom were obese, a minority of patients did not improve after the use of reslizumab, the remaining patients had a reduction in asthma exacerbations, the use of OCS, and rescue medications, and an improvement in lung function.
Mepolizumab	Ortega et al. [201]	Supervised cluster analysis of data from DREAM study of 616 patients with severe asthma	Diminution in exacerbations, significantly greater than non-obese counterparts in other clusters
Mepolizumab	Gibson et al. [202]	Post hoc analysis of data from DREAM, MENSA, SIRIUS, and MUSCA studies (1878 patients with severe asthma)	Reduction in exacerbation rates and improvements in lung function, asthma control, and quality of life, notwithstanding comorbidities, including obesity
Benralizumab	FitzGerald et al. [203]	Analysis of the results from the randomized, double-blind, placebo-controlled SIROCCO and CALIMA studies (2295 patients with severe, uncontrolled asthma)	Reduced annual exacerbations in patients with a BMI > 35 kg/m^2^
Reslizumab vs. mepolizumab vs. benralizumab	Kuruvilla et al. [204]	Retrospective analysis of 58 adults with asthma who were receiving anti-IL-5 therapy	Weight loss was demonstrated after 6 months of all treatments, more pronounced in people with initial BMI > 30 kg/m^2^
Dupilumab	Busse et al. [205]	Randomized, double-blind, placebo-controlled study of 1584 patients with persistent asthma	Reduced the annual rate of asthma exacerbations, regardless of BMI
Tezepelumab	Corren et al. [208] Menzies-Gow et al. [209]	Randomized, double-blind, placebo-controlled trial of 584 subjects with moderate-to-severe asthma and multicenter, randomized, double-blind, placebo-controlled trial of 1061 patients with severe, uncontrolled asthma (12 to 80 years of age)	Reduced the annual rate of exacerbations, blood eosinophils, FeNO, and IgE, and improved FEV1 and quality of life regardless of baseline blood eosinophil level. No data on the effect on obesity-related asthma

**Table 4:** Summary of research findings on other possible treatments for severe asthma.

Therapy	Mode of Action	Study Characteristic	Findings	Author and References
Azithromycin	macrolide antibiotic	randomized, double-blind, placebo-controlled study of 420 patients with symptomatic asthma (213 in the azithromycin group and 207 in the placebo group)	reduced exacerbation rates and improved quality of life in patients with both eosinophilic and non-eosinophilic asthma	Gibson et al. [210]
	macrolide antibiotic	randomized, double-blind, placebo-controlled trial in subjects with severe asthma (55 in the azithromycin group and 54 in the placebo group)	reduced the risk of exacerbations and lower respiratory tract infections in severe non-eosinophilic asthma, improved the quality of life in eosinophilic and non-eosinophilic asthma	Brusselle et al. [211]
Clarythromycin	macrolide antibiotic	randomized, placebo-controlled trial of 45 subjects with severe refractory asthma	reduced neutrophilic inflammation and improved quality of life scores	Simpson et al. [212]
Roflumilast	type 4 cAMP phosphodiesterase inhibitor	double-blind, placebo-controlled, crossover study of 25 subjects with mild allergic asthma	reduced eosinophilic and neutrophilic allergen-induced inflammation	Gauvreau et al. [213]
Roflumilast with montelukast	type 4 cAMP phosphodiesterase inhibitor and leukotriene receptor antagonist	randomized, double-blind, placebo-controlled, multiple-dose, two-sequence, crossover study of 64 patients with uncontrolled mild-to-moderate asthma	improved lung function and symptom control in moderate-to-severe asthma	Bateman et al. [214]
SCH527123	selective CXCR2 receptor antagonist	randomized, double-blind, parallel study of 34 patients with severe asthma	reduced the rate of mild exacerbations and the sputum neutrophil count	Nair et al. [215]
Fevipiprant	non-steroidal prostaglandin D2 receptor antagonist	a systemic review of five articles, including seven randomized trials	increased in FEV1 pre- and post-bronchodilator, did not improve in ACQ score or reduce the number of exacerbations	Jahangir et al. [216]
GB001	selective CRTH2 antagonist	randomized, double-blind, placebo-controlled, dose-ranging, parallel-group, multicenter study of 480 patients with moderate-to-severe asthma with a blood eosinophil count ≥ 250 cells/μL	did not result in a statistically significant improvement in the annual frequency of exacerbations; extended the time to first deterioration; resulted in elevated liver function tests, leading to discontinuation of the treatment	Moss et al. [217]
Golimumab	a human anti-TNF-α antibody	multicenter, randomized, double-blind, placebo-controlled, dose-ranging study of 309 patients with severe and uncontrolled asthma	failed to improve FEV1 or reduce exacerbations; resulted in an unfavorable side effect profile, due to which its use was discontinued	Wenzel et al. [218]
Etanercept	recombinant TNF-α receptor	randomized, parallel, double-blind, placebo-controlled study of 131 subjects with moderate-to-severe persistent asthma	did not improve pred. FEV1, morning PEF, AHR, symptom control, or quality of life, and did not reduce the frequency of exacerbations	Holgate et al. [219]
Brodalumab	human monoclonal antibody against the IL-17 receptor	randomized, double-blind, placebo-controlled study of 302 subjects with inadequately controlled moderate-to-severe asthma	did not improve asthma control and FEV1 in patients with moderate-to-severe uncontrolled asthma who were taking regular inhaled corticosteroids	Busse et al. [220]
Secukinumab	anti-Il-17 antibody	a randomized, double-blind, placebo-controlled study of 46 patients with asthma inadequately controlled with ICS-LABA	did not improve asthma control	Novartis Pharmaceuticals [221]
Risankizumab	monoclonal antibody against IL-23p19	multicenter, randomized, double-blind, placebo-controlled, parallel-group trial of 204 patients with severe asthma	resulted in a shorter time to the first exacerbation	Brightling et al. [222]
Astegolimab	monoclonal antibody against the ST2 receptor	randomized, double-blind, placebo-controlled, dose-ranging study of 502 adults with severe asthma	reduced annual exacerbation rate, increased time to first exacerbation, improved quality of life and lung function	Kelsen et al. [223]
Ipetekimab	monoclonal antibody against IL-33	randomized, double-blind, placebo-controlled study of 296 adults with moderate-to-severe asthma	reduced mean blood eosinophil count, and improved lung function and quality of life	Wechsler et al. [224]
Tralokinumab	human monoclonal antibody against IL-13	randomized, double-blind, parallel-group, placebo-controlled study of participants aged 12–75 years with severe asthma	did not significantly reduce the annual rate of asthma exacerbations (AER) in the all-subject population; improved AER in the high FeNO group	Panettieri et al. [225]
Lebrikizumab	human monoclonal antibody against IL-13	randomized, double-blind, placebo-controlled study of 1081 patients with uncontrolled asthma	an increase in the time to the first exacerbation and a decrease in FeNO were observed in LAVOLTA I and LAVOLTA II; no statistically significant reduction in the frequency of asthma exacerbations was observed.	Hanania et al. [226]
Zweimab and doppelmab	bispecific antibody	study design of monovalent bispecific antibody format, called Zweimab, and a bivalent bispecific antibody, Doppelmab	inhibited Th2-high and Th2-low inflammation due to their targeting of TSLP and IL-13	Venkataramani et al. [227]
Bronchial Thermoplasty	method of reducing ASM hypertrophy	RISA: randomized study of 32 subjects with symptomatic, severe asthma AIR: randomized study of 112 patients with moderate or severe asthma AIR2: randomized, double-blind, sham-controlled study of 297 patients with severe asthma BT10+: follow-up study of 192 participants who were previously enrolled in the AIR, RISA, and AIR2 trials and who had 10 or more years of follow-up since bronchial thermoplasty treatment	Studies showed significant improvements in ACQ and AQLQ and reductions in the use of rescue medications and severe exacerbations; clinical observations regarding lung function were conflicting Assessment of the effects and safety of BT ≥10 years after BT showed a similar incidence of severe exacerbations at the BT10+ visit and one year after the procedure; quality of life and lung function scores at one, five, and ten years after BT were similar.	Pavord et al. [228] Castro et al. [229] Chupp et al. [230] Pavord et al. [231] Thomson et al. [232] Chaudhuri et al. [233]

## Abbreviations

ACD	asthma control days
ACT	Asthma Control Test
ACQ	Asthma Control Questionnaire
AdipoR1	adiponectin receptor 1
AdipoR2	adiponectin receptor 2
AER	annualized rate of exacerbations
AMPK	5’AMP-activated protein kinase
anti-IL-4Rα	interleukin-4 receptor subunit α
ASM	airway smooth muscle
ATDCs	adipose tissue-derived dendritic cells
AQLQ	Asthma Quality of Life Questionnaire
AHR	airway hyperresponsivity
BAL	bronchoalveolar lavage
BMI	body mass index
BT	bronchial thermoplasty
cAMP	cyclic adenosine monophosphate
CD4	glycoprotein cluster of differentiation 4
CPAP	continuous positive airway pressure
CRS	chronic rhinosinusitis
CRTH2	chemoattractant-receptor homologous molecule
CXCR2	chemokine 8 receptor-2
DM	diabetes mellitus
DMt2	diabetes mellitus type 2
ED	emergency department
EOA	early-onset asthma
ERV	expiratory reserve volume
FeNO	fractional exhaled nitric oxide
FEV1	forced expiratory volume in one second
FP/SAL	fluticasone propionate/salmeterol
FRC	functional residual capacity
FVC	forced vital capacity
GCS	glucocorticosteroid
GERD	gastroesophageal reflux disease
GINA	Global Initiative for Asthma
GLP-1	glucagon-like peptide-1
GLP-1R	glucagon-like peptide-1 receptor
GLUT	glucose transport proteins
GR-α	glucocorticoid-alpha receptor
GR-β	glucocorticoid-beta receptor
Hb1AC	glycated hemoglobin
HDL	high-density lipoprotein
HRCT	high-resolution computer tomography
ICS	inhaled corticosteroid
ICU	intensive unit care
IgE	immunoglobulin E
IL	interleukin
LABA	long-acting beta-2 agonists
LAMA	long-acting antimuscarinic
LOA	late-onset asthma
M1, M2	macrophage subpopulations 1 and 2
MAP	mitogen-activated protein
MCP-1	monocyte chemoattractant protein-1
MetS	metabolic syndrome
MUC5B	mucin 5B protein
NIH	National Institutes of Health
NK	natural killer cell
NF-κB	nuclear factor kappa-light-chain-enhancer of activated B cells
NLRP3	inflammasome of nucleotide oligomerization domain-like receptor protein 3
OCS	oral corticosteroid
OR	odds ratio
OSA	obstructive sleep apnea
PAI-1	plasminogen activator inhibitor-1
PBMC	peripheral blood mononuclear cells
PEF	peak expiratory flow
PGA	paucigranulocytic asthma
PPI	proton pump inhibitor
ROS	reactive oxygen species
RV	residual volume
SABA	short-acting beta-agonists
sHDL	serum high-density lipoprotein
SPT	skin prick test
ST2 receptor	interleukin 1 receptor-like 2
Tc	cytotoxic T cell
TG	triglyceride
TGF-β1	transforming growth factor β
Th	T-helper cell
TLC	total lung capacity
TLR	toll-like receptor
TNF-α	tumor necrosis factor α
*t*PA	tissue plasminogen activator (*t*PA) and urokinase (uPA)
Treg	T regulatory cell
TSLP	thymic stromal lymphopoietin
uPA	urokinase plasminogen activator
VC	vital capacity
WHO	World Health Organization
